# Men’s perceptions of prostate cancer diagnosis and care: insights from qualitative interviews in Victoria, Australia

**DOI:** 10.1186/s12885-017-3699-1

**Published:** 2017-10-27

**Authors:** Maggie Kirkman, Kate Young, Susan Evans, Jeremy Millar, Jane Fisher, Danielle Mazza, Rasa Ruseckaite

**Affiliations:** 1 Jean Hailes Research Unit, School of Public Health and Preventive Medicine, Faculty of Medicine Nursing and Health Sciences, Monash University, 553 St Kilda Road, Melbourne, VIC 3004 Australia; 2 Department of Epidemiology and Preventive Medicine, School of Public Health and Preventive Medicine, Faculty of Medicine Nursing and Health Sciences, Monash University, Melbourne, VIC Australia; 3 Radiation Oncology, Alfred Health, Melbourne, VIC Australia; 4 Department of General Practice, School of Primary Health Care, Faculty of Medicine Nursing and Health Sciences, Monash University, Melbourne, VIC Australia

**Keywords:** Prostate cancer, Qualitative research, Men’s health, Australia

## Abstract

**Background:**

The Victorian Prostate Cancer Registry (Australia) revealed poorer rates of survival for men diagnosed with prostate cancer in one Victorian regional area than for men in metropolitan Melbourne. We sought to explore the perceptions and experiences of prostate cancer diagnosis, treatment, and care of men diagnosed with prostate cancer who lived in regional or metropolitan areas and of men who had not been so diagnosed. Our goal was to contribute to the evidence from which can be built continuing improvements in prostate health care.

**Methods:**

Using the qualitative method of in-depth interviews to gain access to explanation and meaning, we interviewed 21 men: 10 recruited through the Prostate Cancer Outcome Registry-Victoria and 11 from the community. Transcripts were analysed thematically.

**Results:**

We identified four main themes within which men discussed prostate cancer: Case-finding, Diagnosis, Treatment and Care, and Spreading the Word. Contrasts revealed between regional and metropolitan areas related mostly to the more limited supportive care in regional areas.

**Conclusions:**

It is evident from the perspectives of these men that every aspect of prostate cancer care would benefit from attention: publicising the need to check prostate health, treatment, and supporting men in the years after treatment. Continuing to work on systemic improvements is an important goal for all those committed to men’s health.

**Electronic supplementary material:**

The online version of this article (10.1186/s12885-017-3699-1) contains supplementary material, which is available to authorized users.

## Background

There is a good chance of long-term survival after a diagnosis of prostate cancer, especially in countries like Australia with a comprehensive healthcare system [1]. Nevertheless, inequalities are evident, with better prognoses for men who live in Australian metropolitan areas than in rural or regional areas [2]. (Definitions vary, but metropolitan areas in Australia are within the major capital cities of Sydney, Melbourne, Brisbane, Perth, Adelaide, and Canberra and their suburbs; regional areas are usually taken to be towns and small cities outside the major capital cities; and rural areas are what remain [3, 4].) Similarly, men in rural areas in New Zealand were found to be less likely to participate in prostate-specific antigen (PSA) screening and to be diagnosed with prostate cancer at a later stage than men in metropolitan areas [5]. This disparity is not confined to the southern hemisphere; higher death rates and incidence of late-stage disease, as well as lower prevalence of PSA screening, were found in non-metropolitan areas than metropolitan areas of the United States [6]. Disparities are not solely geographic; Australian male Vietnam veterans have twice the incidence of prostate cancer than civilian men, but both categories were found to have inadequate knowledge of detection and few had discussed the topic with their GPs [7]. After establishing the Victorian Prostate Cancer Registry [8], it became evident that there were differences in care and treatment outcomes for men diagnosed with prostate cancer between one regional area in the state of Victoria and metropolitan Melbourne (Victoria’s capital city) [9]. Men in the region are more likely to be diagnosed at an older age with more advanced disease and to have a longer time interval between diagnosis and treatment, resulting in a five-year survival rate of 86%, compared with 93% survival of men in Melbourne.

It is difficult to untangle potential reasons for the higher cancer-related mortality rates in rural and regional than urban areas and for other observed discrepancies. Evidence that enables us to understand aspects of men’s experiences of prostate cancer might contribute to improving service delivery and care that can be extended to work aimed at reducing inequity. We have the puzzling statistics; what do we know about the psychosocial aspects of prostate cancer?

Research tends to focus on men who have been diagnosed, especially on symptoms and the side-effects of treatment. It is commonly found that disruption to quality of life after treatment troubles men, their partners, and physicians [10, 11]. Men may regret their treatment because of side effects, especially sexual and urinary dysfunction [12]. Qualitative interviews with 10 men after radical prostatectomy revealed that they were distressed and anxious and yearned for the return of their pre-diagnosis life [13]. In the year after diagnosis, men continue to report unmet needs for supportive care [14]. Some men, however, choose to cope on their own with their anxiety and distress after radical prostatectomy, often in relation to incontinence and erectile dysfunction [13]. A qualitative systematic review of men’s experience of and need for supportive care found that the most valued form of support men experienced following diagnosis was from peers one-to-one and from partners [15].

‘Masculinised’ supportive care, especially in the form of exercise, has been advocated because of the potential for the emasculating effects of treatment to compromise men’s mental health [16]. Fear of stigma was also found in interviews with men who said that they rarely disclosed their prostate cancer to anyone but their wives and minimised the need for support [17]. These interviews revealed, too, that the emotional challenges of managing a diagnosis of prostate cancer and its treatment can be difficult not only for men but also for their partners [18, 19]. More recently, an online survey found that, when men recovering from prostate cancer and its treatment are in a relationship, there is interaction between the men’s mood and the quality of the relationship [20].

Despite the fact that prostate cancer is the most commonly diagnosed tumour reported to cancer registries in Australia [21] and the second most common cancer in men globally [22], men in Australia often do not raise the topic with their GP, even when they are aware of a family history [23]. GPs are pivotal to diagnosis and care of men with prostate cancer. Nevertheless, the Royal Australian College of General Practitioners does not recommend screening for prostate cancer unless a man requests it [23]. GPs in Australia who responded to a web-based questionnaire revealed widely diverse approaches to testing, which the authors attribute predominantly to the absence of clear and consistent guidelines [24]; our own interviews with GPs found similar concerns about inconsistent guidelines [25]. These GPs found it hard to explain differences in outcomes in regional and metropolitan areas apart from referral patterns and a belief that men in regional areas were less likely to visit a GP [25].

In order to reach a deeper understanding, we sought to explore the perceptions and experiences of prostate cancer diagnosis, treatment, and care among men diagnosed with prostate cancer who lived in either a regional or a metropolitan area. To illuminate experiences that might precede or impede diagnosis, we included men who had not been diagnosed with prostate cancer and who lived in similar areas, with the expectation that they would reflect the views of men in the general community. Our objective was to learn from men any aspect of their experiences or reflections that could inform what we know about the association of geographic areas with diverse outcomes in prostate cancer. Our ultimate goal was to add our findings to our continuing program of research designed to contribute to improvements in prostate health care.

## Methods

To address our research aims of seeking explanation and meaning, we adopted a qualitative approach. Qualitative methods have an important role in health research in enabling access to the meaning of disease for those who are experiencing it and others to whom it is relevant [26].

### Eligibility and recruitment

All men diagnosed with prostate cancer in Victoria are reported to the Victorian Cancer Registry. If men give informed consent, their details are also listed on the Prostate Cancer Outcome Registry-Victoria (PCOR-V) [8]. Men on the PCOR-V were eligible to participate if they had been given a pathological diagnosis of prostate cancer in 2013 and lived in a regional area of Victoria or metropolitan Melbourne. Men who had not been diagnosed with prostate cancer were eligible to participate if they lived in a regional area of Victoria or metropolitan Melbourne. Men on the Victorian Prostate Cancer Registry were notified of the research by letter and invited to volunteer; men of similar ages without a history of prostate cancer were encouraged to volunteer through an advertising and media campaign in regional Victoria and metropolitan Melbourne. Volunteers gave informed consent after reading a detailed explanation of the research. All participants were offered $20 gift vouchers.

### Data collection

In-depth qualitative interviews were conducted by telephone from April to July 2015 in Victoria, Australia, by a member of the research team (KY) experienced in in-depth interviewing. Men who chose not to send a signed consent form gave (recorded) oral consent before the interview. Each interview began with a request to describe ‘your experiences of prostate cancer’ (men who had been diagnosed) or to describe ‘anything at all ... that you have experienced of prostate cancer’ (men who had not been so diagnosed). The interviewer pursued matters identified by the men as important, with prompts, if necessary, that included consulting a GP, the process of diagnosis, and medical care. At the conclusion of the interview, men were asked what advice they would give to other men, doctors, and policy makers about prostate health. Each man interviewed was invited to choose a pseudonym by which he would be known in any publications. The interview guides are available as Additional files 1 and 2.

### Data management and analysis

Interviews were audio-recorded (with permission) and transcribed. Identifying details were deleted from the transcripts before thematic analysis [27] began. After familiarising ourselves with the data, we followed the usual iterative process of moving between single transcripts and the whole data set and between emerging themes and the developing thematic structure. Four members (MK, KY, JF, and RR) of the research team identified themes in each transcript and negotiated and finalised the emerging thematic structure. Transcripts were searched again to ensure that the analysis was accurate and comprehensive; the revised scheme was applied to each transcript and exemplary quotations were selected. The software program NVivo [28] was used to help manage the data.

### Ethics approval

Approval to conduct this research was granted by the Monash University Human Research Ethics Committee (#CF15/858–2015000385).

## Results

### Participants

In-depth interviews were conducted with 10 men who had been diagnosed with prostate cancer, six of whom lived in metropolitan Melbourne and four in regional Victoria. They were aged from 56 to 81 years (mean 69); their age at diagnosis was 53 to 79 (mean 67). All but three men had private health insurance at the time of diagnosis. Four of the men were in paid employment; the rest had retired. Seven men were in a relationship; three were single. Six of the 10 men had been born in Australia. There were also 11 men without a history of prostate cancer. Seven of these men lived in metropolitan Melbourne and four in regional Victoria. They were aged from 44 to 78 years (mean 63). Seven of the men were in paid employment; four had retired. Eight men were in a relationship; three were single. Eight of the 11 men had been born in Australia.

Interviews lasted, on average, about 30 min with men with a prostate cancer diagnosis (37 min regional, 27 min metropolitan) and about 15 min for men without such a diagnosis (equal for regional and metropolitan). We felt that this was a long time for men to discuss the single, sensitive topic of their prostate, whether or not they had been diagnosed with cancer. One man with a cancer diagnosis said, for example,

*I’ve talked more about me prostate episode now than I have talked to anybody.*

- Harry, regional, 74 years, prostate cancer

### Themes

Four main themes were identified from the transcripts: Case-finding, Diagnosis, Treatment and Care, and Spreading the Word; each of these had sub-themes, detailed below. Men revealed much more about their experiences with and understanding of prostate cancer care than potential differences occasioned by their place of residence.

In what follows, quotations from diagnosed and undiagnosed men are distinguished by the presence or absence of ‘prostate cancer’ following the pseudonym, type of residential area, and age after each quotation.

### Case-finding

We apply the term ‘case-finding’ to the detection of asymptomatic early prostate cancer and reserve ‘screening’ for a systematic program of national population testing designed as a cost-effective means of reducing deaths from prostate cancer.

#### Awareness

Men were generally aware that prostate cancer is an important problem for men’s health. Men who had and had not been diagnosed said that it was good to have well-known men talking in the media about their experience of prostate cancer; the publicity contributed to creating awareness and prompting men to inquire about case-finding tests when they next saw a GP:

*There’s been personalities on TV that have had the operation and I think that’s one of the things that inspired me: ‘Hang on, this guy’s got it. Perhaps I should go for a check-up’.*

– Ian, metropolitan, 70 years, prostate cancer

However, at least one man (without prostate cancer) thought that awareness was inadequate and information scarce:

*There’s certainly not an education program out there about symptoms of prostate cancer. No-one knows what they are, or you’re certainly not told about them in any shape or form in terms of, you know, ‘These are the symptoms that might lead you to believe you that you might have an enlarged prostate or prostate cancer’. No-one knows that, or I’ve certainly never seen it.*

– Andrew, metropolitan, 65 years

As a result, Andrew was not ready to present himself for testing: *‘If someone could tell me definitively that you’re going to die from it then, yes, I might do something about it’.*

Undiagnosed men discussed ways in which they thought information about prostate cancer should be distributed to enhance awareness. Newspaper articles and advertisements were often mentioned, along with written information provided by their GP. Although the internet is available throughout Victoria, access speed is acceptable in the relevant regional area, and its use was taken for granted by most participants, metropolitan men more often spoke about using the internet to find health information. One regional man in his seventies who had not been diagnosed with prostate cancer said that he did not use the internet:

*I don’t have online. So I suppose I’d have to see it in the paper, or—I don’t know how much they like to put in the paper about it, or whether I’d like information, but I presume there is information available if you ask, I suppose.*

– Desmond, regional, 74 years

Desmond may be in a minority of rural and regional men who choose not to use the internet, but his inclusion in this research is a reminder that a variety of approaches need to be employed in order to improve awareness of prostate cancer.

Brief television campaigns were also favoured by metropolitan men:

*It would be better advised to do a refresh advertising but make it meaningful and have it on for a short period of time and then that’s it, finish it. Because otherwise, especially guys, I think, tend to turn off.*

– Adam B, metropolitan, 55 years

Some of the regional men spoke of attending fundraising events for prostate cancer; they found them to be enjoyable and educational experiences:

*They get people together, and I think it opens up people’s minds about what problems there could be there.*

- Robert, regional, 78 years

#### Facilitators of case-finding

There were notable differences in the circumstances surrounding men’s case-finding experiences across metropolitan and regional areas. Regardless of diagnosis status, men who lived in metropolitan areas typically underwent a prostate cancer case-finding test during a general medical check. In contrast, men who lived in a regional area and who had been diagnosed reported undergoing a case-finding test at the recommendation of their doctor while receiving care for another health problem:

*If you’ve had pre-existing health problems, you naturally go to the doctor more than you would otherwise. … The more often you go to the doctor, the more often he’ll look and sort of say, ‘Oh, you need a PSA’.*

– Malcolm, regional, 77 years, prostate cancer

Men who had not been diagnosed and lived in a regional area mostly reported requesting a case-finding test from their GP after becoming aware of the potential benefits of doing so from men they knew who had prostate cancer, or after attending a prostate cancer fundraising event:

*I just requested it. I just thought, well, because of my age and everything, I’ve never had the tests before, and I thought, ‘Oh, I’ve probably been lucky so far. It’s time to go ahead and really get the medical background of it now.’*

– Robert, regional, 78 years

One of the men reported having a case-finding test during an annual check-up at his workplace. He thought it would be a useful way for other men to be screened:

*It prompted me to do something and it was onsite, obviously. You didn’t have to go out of your way or anything.*

– Nick, regional, 47 years

Some undiagnosed men, particularly those from a metropolitan area, perceived their GP as reluctant to conduct prostate cancer case-finding tests. Two men (one regional, one metropolitan) reported that GPs typically didn’t initiate PSA testing. For example:

*They [GPs] usually don’t offer a PSA test; you have to ask for it.*

- Pinky, regional, 59 years

Another two metropolitan men were unhappy when their GP delayed their case-finding tests because *‘recommendations were changing’* or when they refused to perform digital tests; for example:

*I’m disappointed that he [GP] doesn’t want to do the finger test. He wants to refer me to a urologist, so I thought doctors are supposed to encourage men to not be embarrassed about it, and this is the other way around. He says, ‘Oh, look, the PSA should be enough’. But I happen to know that PSA and finger test alone is not enough. You need—even both is not 100 per cent guaranteed, but having both is fairly safe. But I’m disappointed that he’s one of those that doesn’t want to do it. I thought he’s supposed to encourage men to do this, and not disappoint them.*

– Luke, metropolitan, 66 years

### Diagnosis

#### Process of diagnosis

Interviews illuminated aspects of experiencing diagnostic procedures; two men spoke about undergoing a biopsy as being among the most difficult aspects of their prostate cancer experience. Ian (metropolitan, 70 years, prostate cancer) said *‘you don’t know what’s going on’* because there is inadequate support until after the diagnosis. According to John, in the waiting room, men were treated *‘like a bunch of cattle’* and, with no anaesthetic, the procedure was painful and uncomfortable. He told of receiving no follow-up care:

*He said he would ring me in a couple of days. A week went by. I rang the consulting practice and asked what was going on. … I was not very happy with that, because I felt that anybody facing a possible cancer is going to have high stress levels and to leave me sitting like a shag on a rock was not very good.*

– John, regional, 56 years, prostate cancer

#### Time to diagnosis

Men living in metropolitan and in regional areas who had been diagnosed with prostate cancer recalled having to wait what seemed like a long time for a diagnosis, but differed in what this meant to them. Men from metropolitan areas said that the wait was made bearable by the support of their healthcare providers. There were also comments about the effect of different coping styles. For example, Ben (metropolitan, 61 years, prostate cancer) said he was *‘good at putting things in a box’* whereas he thought others may struggle with *‘periods of uncertainty’.* In contrast to men from metropolitan areas, men living in regional areas found the wait to be difficult, especially because they lacked support:

*I think the worst part of the whole affair was waiting from the time I had my biopsy ‘til the time I was told that I had prostate cancer. It was in between interviews. Like, you know, your next interview with the urologist was a month’s time, so that was—or over a month, I think it was—so that was terrible, because your imagination plays havoc with you, and you’re not up to the stage of having the support or the acceptance that you have it, and that’s it not too bad anyway, sort of thing.*

– Malcolm, regional, 77 years, prostate cancer

### Treatment and care

#### Decision making

Men diagnosed with prostate cancer described the circumstances under which they chose to have certain treatments. There were notable differences in the responses of men from metropolitan areas compared with men from regional areas. The former spoke of a careful decision-making process that involved *‘working together’* with their doctor. Men from metropolitan areas also said they relied on various sources of information, including what was given to them by their doctor in written form and on DVD, the internet (the Prostate Cancer Foundation website was cited), and discussions with their family. These men particularly valued learning about the experiences of other people who had been diagnosed with cancer, not limited to prostate cancer:

*Whilst there was plenty of reading matter out there, … it’s finding people that have been through it that I found of value. … Like you can read and read and read but, at the end of the day, it’s talking to somebody, I think, is the most important part.*

– Ian, metropolitan, 70 years, prostate cancer

One man said it was important to have a good relationship with one’s healthcare providers in order to decipher the contradictory information available online:

*I think, for the ordinary bloke in the street—with people having arguments about population-level screening as opposed to individual patient management, as it were, and the interpretation of results, it’s very difficult for the average person to make much sense of it, so that makes the individual relationship with the GP and the specialist so important, because you just have to trust them to interpret the information and say what’s best for you.*

– Ben, metropolitan, 61 years, prostate cancer

In contrast, men living in regional areas tended to report making a decision about treatment *‘on the spot’,* at the time of diagnosis or soon after, based on their doctor’s advice alone.

*When [the specialist] recommended having non-nerve-sparing surgery, in terms of the actual decision to do the surgery, [my wife] and I, we made the decision on the spot, like, ‘Yes, we will do that’. I did get cold feet after it, and I rang the [specialist] to run through the whole process again beforehand, and re-affirm to me that that was why he felt it was the best option.*

– John, regional, 56, prostate cancer

Some men in regional areas recalled being given only limited information. John said he had consulted websites after *‘being very dissatisfied’* with information given him by his specialist:

*The process to change, swap urologists was [word unclear] slow and took a while, and it was just through dissatisfaction, with a sense that we weren’t getting, we weren’t being told enough.*

– John, regional, 56, prostate cancer

#### Treatment side effects and quality of life

Men who had been diagnosed with prostate cancer spoke about the side effects of their treatments; some men who had not been diagnosed also revealed awareness of side effects: hormonal symptoms (such as hot flushes), problems with the bowel and bladder (predominantly incontinence), and difficulties with sexual function.

*The side effects of the hormone treatment have probably been the worst thing to handle. I have had after-effects, and I believe that it’s probably through the radiation. I have bowel problems. I will go to the toilet and use my bowels first thing in the morning, and then within five or ten minutes, I’ve got to race back again, sometimes three times, so that didn’t happen before.*

– Malcolm, regional, 77 years, prostate cancer

There were differences in the way that men described their experiences of side-effects, apparently related to where they lived. Men from a metropolitan area typically presented their experience of treatment side effects as steps towards the goal of a return to health. Although it was initially *‘devastating’* to experience the symptoms, most metropolitan men emphasised the process of improvement and were *‘quite excited’* about eventually being symptom-free. In contrast, regional men’s accounts centred more on the theme of acceptance:

*So there are those risks in it [the treatment]. With anything, I think you’ve just got to face up to it*.

- Malcolm, regional, 77 years, prostate cancer

In general, men who experienced reduced sexual function expressed disappointment but gave priority to their survival:

*It’s important, but I mean if you can’t have it, you can’t have it. … It’s better to be alive than die and have that*.

- Domenico, metropolitan, 81 years, prostate cancer

Two metropolitan men described their experiences of seeking care for sexual symptoms. Ben (metropolitan, 61 years, prostate cancer) spoke to a surgeon who encouraged him to *‘give it plenty of time’* before he considered a penile implant; Ben eventually had the implant which he described as *‘very, very successful’*. Frank (metropolitan, 74 years, prostate cancer) reported being offered surgery by his specialist who said he wanted him to *‘live like a man’,* but Frank said that he did not *‘need that [surgery] at the moment’.*

Only men from regional areas spoke of the moderating effect of their partner’s support on their experiences of sexual dysfunction:

*He [the doctor] said, ‘You probably won’t be able to have an erection for about 10 years after this hormone treatment’. And she immediately said to him, ‘We’ve learned to hold hands in bed.’ … You know, there is life after 70, anyway.*

– Malcolm, regional, 77 years, prostate cancer

*So look, we certainly went on with it, and had sex fairly regularly. It sort of changed the way—nothing can recover what you had in the past, so it was a very difficult period. … We’ve [wife and I] learned tricks and ways to use it [vacuum pump], and we joke about it, and I’d say that we’re making the best of a bad lot.*

– John, regional, 56 years, prostate cancer

Although men without prostate cancer who lived in a regional area did not speak about their perceptions of treatment side-effects, those living in the metropolitan area did. They reported hearing that those who had experienced prostate cancer *‘wonder whether the benefit of the treatment outweighs the loss of functionality’* (David, metropolitan, 69 years) and said that they would find the side effects to be *‘devastating’* (Luke, metropolitan, 66 years), particularly sexual dysfunction.

#### Health care

##### GP Care

All men spoke about what they did and did not like about various aspects of the care they had received from GPs; there was little difference in their comments except that men living in metropolitan regions described this in greater detail. Men valued GPs whom they perceived as being ‘proactive’ about specific health matters rather than taking a ‘wait and see’ approach, and who were ‘reliable’, ‘careful’, ‘professional’, and a ‘good listener’. They also liked their GPs to take the health of their older patients seriously:

*He said, ‘A man of your age, you can forget about it, and you’ll live another eight to ten years’. And I said, ‘Wake up to your bloody self! It’s cancer!’ I said, ‘Eff it off!’ ... And, you know, wait until that man [doctor] gets to his seventies and somebody tells him that. … My mother lived to 96; I want to do the same.*

- Harry, regional, 74 years, prostate cancer

Men appreciated doctors who conducted regular case-finding tests and monitored their general health at each visit:

*‘Hello, get on the scales’, and while he [GP] was talking he’d take your blood pressure. To me, that should be part of most GP, most consultations.*

– Malcolm, regional, 77 years, prostate cancer

Some men said that they were not told the results of their tests; two of them elaborated on how they felt about it. Bob said:

*I’ll go to the effort of going to some place to get some scans or whatever done, and then I won’t hear back from the clinic. … That’s probably definitely an area they could improve: actually getting back to the patient instead of leaving them in limbo.*

– Bob, metropolitan, 44 years

However, Robert (regional, 78 years) was not concerned about not receiving his results because he knew the doctor and was *‘sure he would have contacted me if it had been anything at fault there’.* Some men selected their GP or GP practice because they were near their home or work, and then would stay with the one GP or practice for years, valuing the chance to build a *‘good relationship’*.

A few men described how important their partners were in helping them to navigate medical care in general:

*[My wife]‘s been just totally supportive. She has attended all my major appointments with me. Sometimes I miss points in talking to people, or don’t pick up some of the nuances or the subtleties of what they’re telling me. It’s handy having her there, that she picks that up as well. This is as a result of my stroke. … She’s been very helpful from that point of view as a second person to repeat or to talk about the things that we’ve discussed with the specialist.*

– Adam A, metropolitan, 70 years

Men varied in their attitudes to medical care. For example, Adam B (metropolitan, 55 years) said that he found the *‘whole medical experience a little bit intimidating’* and appreciated his GP putting him *‘more at ease’* because *‘you can relate to him, and he relates to you as well’*. Malcolm (regional, 77 years, prostate cancer) said he has *‘never been worried about going to the doctor’* because he has a history of health problems that exposed him to the medical setting from a young age.

It was common to hear men say that men in general *‘don’t go to the doctor unless they’re dying’* (David, metropolitan, 69 years). Some men thought this tendency was diminishing while others asserted it as an immutable fact. When the interviewer asked such men how they explained their own regular medical checks and that other men in their lives did the same, they claimed to be the *‘exception’*.

##### Hospital Care

Some men who had been treated for prostate cancer appreciated the care they had received in hospital; this did not differ by their area of residence. No man reported an adverse experience of hospital care, apparently choosing to emphasise what mattered to them most: their survival. The kindness and attentiveness of nurses and administration staff were frequently mentioned as contributing to good hospital experiences:

*Oh, his nurses, I mean they are also—. … They appreciate, you know? Like a nurse, you can open your heart, ja? And they were helpful in any possible way.*

*–* Frank, metropolitan, 74 years, prostate cancer

*I got a bit depressed at times, but every time I went there [to the hospital], you were buoyed up. … [Once I settled in with my specialist], anyone that I dealt with up at the [regional hospital] in the [cancer clinic], were just so laid back and happy, even the lady who is in charge of the—she was the cleaner, actually, but she would also make cups of tea if you wanted one, and, you know, wander in and tell jokes. She was—that type of person was like a very, very important person in the organisation, even though she was just a cleaner or a house lady, because she was the front person. You know, she was the person you saw more. … When it finished, I really started to miss it, because the type of, the attitude of the staff at the [cancer clinic], particularly a couple of the operators, was excellent.*

– Malcolm, regional, 77 years, prostate cancer

The opportunity to talk with other men in hospital could make a difference. John (regional, 56 years, prostate cancer) said that sharing a hospital room with another man who had also been diagnosed with prostate cancer was *‘a really lovely time’* because he *‘bonded’* with this man over their common circumstances.

##### Mental Health

A few of the men who had been diagnosed with prostate cancer spoke briefly about mental health care within overall prostate cancer care. For example, Ben (metropolitan, 61 years, prostate cancer) said that he relied on his GP or specialist to pick up the ‘slight signs’ that he needed support for his mental health but believed his specialist would be less able to do this because he is ‘highly focused on the mechanics’ (the physical aspects). Two men from regional areas reported diverse experiences. Harry (regional, 74 years, prostate cancer) said that he was not offered any mental health support but it would not have been necessary because he had ‘good friends around me to help me whenever I need it’. John (regional, 56 years, prostate cancer) recalled seeing a psychologist but thought that he ‘didn’t really get much out of it’. John had experienced care in both the regional area in which he lived and in a metropolitan facility and found the latter to be far more beneficial to his mental health:

*The difference in caring between the [regional] practice and the [metropolitan] practice was profound. … [In the regional practice] there was just a complete lack of understanding as to what I was going through.*

– John, regional, 56 years, prostate cancer

One man spoke of the need to take care of his own mental health:

*I socialise with a lot, a group of Australians; they’re elderly people. I’m going out four times a week, really. If I don’t feel well, we just have a social get-together. We play cards, we have a cup of coffee about 10 o’clock to 10.30. Sometimes they have a social together and they have a bit of a band, and we’re all rolling around the dancing floor. Everybody reckons we’re dancing, but we pass the time, and that’s good. Instead of staying home on your own, it’s—if you’re on your own, especially if you don’t feel well, it’s the worst. You feel a lot worse.*

- Domenico, metropolitan, 81 years, prostate cancer

While the men did not speak about mental health in detail, the inference to be drawn from their more general comments was that support for mental health should be integrated into all levels of care. Rather than allocating responsibility for mental health to one or two professions, such as psychology or social work, maximum benefit would be gained were all healthcare providers to be sensitive to the psychological and emotional challenges presented by a diagnosis of prostate cancer and its treatment.

### Spreading the word

#### Talking to other men

All interviewees commented on whether men talked about their experience of prostate cancer or discussed general men’s health topics with each other. Men who had been diagnosed with prostate cancer raised similar matters regardless of where they lived. Men who had not been diagnosed with prostate cancer, particularly those living in a metropolitan area, said they thought that men did not discuss prostate cancer or other health matters. Bob (metropolitan, 44 years) was adamant that men talk only about *‘sport and fishing and stuff like that’*, despite, only moments before, having described discussing his employer’s experience of prostate cancer with him. Most of the undiagnosed men said that it was important to communicate with their sons about the need to take care of one’s health:

*We can talk about that [men’s health], yeah. He’s overseas now but he encourages me to keep myself checked.*

– Luke, metropolitan, 66 years

Men who had been diagnosed with prostate cancer commented on the stereotype of men’s supposed inability to discuss such *‘personal’* topics with one another. They often reported actively challenging these attitudes by discussing their experience of prostate cancer with any men willing to listen (most of whom did), particularly at informal barbecues and fundraising or awareness events:

*I think I’ve been helping him [friend with prostate cancer] with it because I’ve been talking very openly about it … He’s a very popular chap and he talked about his prostate cancer that he’s had, and that he’s still having the treatment. ‘But look at Malcolm. He’s sitting down there, and [name] up the back, he’s got it too’, and it was a very open discussion. … That’s a good attitude to have rather than not talk about it.*

– Malcolm, regional, 77 years, prostate cancer

*All my friends, I tell them to make sure they have a blood test every 12 months. Yeah, it’s a must for anybody over 50, I’d say. But then again, younger men can get prostate cancer as well, but as you get older—. I seen a program on TV a few years back, and they said that every man alive will get prostate cancer, and if he doesn’t die of a heart attack, a stroke, or an accident, prostate cancer will kill him, unless it’s diagnosed and fixed up. So that told me I’ve got to keep an eye on it.*

- Harry, regional, 74 years, prostate cancer

#### Advice to men

When participants were asked if they had any advice for men about prostate health, their responses were consistent regardless of diagnosis or region of residence. They said that men should be tested (using the PSA test) during an annual medical check-up with their GP. Some recommended an initial test that would establish a *‘baseline position’* for comparison in subsequent years. Men who had been diagnosed with prostate cancer made comments consistent with advice from Ben (metropolitan, 61 years, prostate cancer): they would encourage others to *‘take it seriously. Don’t panic, but take it seriously’.*

## Discussion

Contrasts between regional and metropolitan areas indicated by the participants related mostly to the more limited supportive care available in regional areas. Our participants acknowledge that much of this responsibility for supportive care falls to GPs, who not only manage discussions about case-finding and treatment but also coordinate continuing care and psychosocial support. They would like GPs to take the initiative in preventive medicine and not to dismiss men’s health concerns because they perceive the men as old. It may be worth reconsidering GPs’ role in introducing the topic of prostate cancer, given men’s preferences and the identified tendency of men not to raise the topic with their GPs [23]. Participants’ concerns about guidelines and case-finding are supported by evidence that variability in clinical guidelines in Australia and overseas may contribute to GPs’ inconsistent screening techniques [24, 29, 30]. GPs will need to ensure that their patients understand that the most recent guidelines recommend that digital rectal examinations are undertaken by specialists and not GPs [31, 32].

Given men’s reported distress while they waited for a diagnosis, attempts should be made to monitor and minimise the waiting times between biopsy and initial consultation. This is particularly challenging in regional areas, where specialists’ visits are often infrequent. Strategies for dealing with time to diagnosis must incorporate the need to mitigate the adverse psychological consequences of this stressful event. Individual clinicians are unlikely to be able to control waiting times; this is a matter for systemic action. However, coordination between GPs and specialists and developing means of checking on patients’ mental state and providing psychological care when necessary would contribute to minimising men’s distress.

Support that recognises challenges to the mental health of men with prostate cancer [16] and contributes to wellbeing may best come from a team with skills in mental health care as well as physical. The inclusion of cancer specialist nurses in the team, care tailored to individual needs, and psychosexual support are likely to promote wellbeing [15]. Care for mental health may need to be extended to encouraging social engagement with the community, not necessarily limited to men with prostate cancer but bringing together people with shared interests (for example, dancing, a community-based ‘men’s shed’ [33]).

Men’s comments about the valued support of their partners is consistent with evidence of the interaction between quality of relationship and men’s mood [20]; consideration may need to be given to including men’s partners in consultations, if the man agrees. Our results concur with other findings that, in addition to the support of their partners, men value peer support [14, 15]. It may be beneficial to make opportunities for men diagnosed with prostate cancer to talk with peers similarly diagnosed contemporaneously or in the past.

Although participants asserted that men did not discuss their symptoms of ill-health and avoided visits to the doctor—a common masculine stereotype [34–36]—the stereotype was not evident in the participants’ accounts of themselves. This could be considered participant bias, an inevitable outcome of using volunteers (who can be assumed to be interested in the research topic) rather than taking the unethical and impermissible route of compelling research participation. Similarly, we did not find fear of stigma, in contrast to others [17]; we found men ready to talk publicly. It is possible that public health campaigns may have reduced the stigmatising effects of diagnosis, although erectile dysfunction may still be problematic. It is possible that men who volunteer for research on prostate cancer when they have not themselves been diagnosed may be more aware of prostate cancer than men who do not volunteer. Another limitation is that, despite taking care not to assume heterosexuality in our participants, we do not appear to have recruited men who identified any other way. We recognise that gay men’s experiences of prostate cancer may differ from straight men’s experiences [37] in ways we cannot specify from our evidence.

Men made it clear that they need information at all stages of their encounter with prostate cancer. The disparity that has been found between the way that men with prostate cancer and healthcare professionals assess men’s information needs and preferences underscores the need for communication between doctors and their patients [38].

Participants’ reflections have implications for health care that are consistent with previous research. Although universal screening is generally not recommended [31, 32], prostate cancer must be detected early in order to enable the best outcome; it is in keeping with this goal that men urged using diverse means of maintaining awareness in the community, both state-wide (such as using well-known men in TV advertisements) and locally (gatherings around a barbecue). Awareness of the need to check prostate health should take place in a healthcare environment that considers men’s needs from being tested, through diagnosis and treatment, to living with the physical and psychological after-effects. Such supportive care includes multidisciplinary teams [39], the members of which communicate with each other and the patient [38], enabling men to be confident that their care is coordinated and not disjointed. Supportive care is not, however, the province solely of health professionals but should be practised by all staff who come in contact with patients, including receptionists, orderlies, and cleaners. The practice of sensitivity, consideration, and professional warmth can make valuable contributions to patients’ health and wellbeing.

## Conclusions

It is evident from the perspective of these men that every aspect of prostate cancer care would benefit from attention, beginning with publicising the need to check prostate health and continuing through the whole process to supporting men in the years after treatment. A weak link at any point would undermine the whole. A program to normalise and publicise the need to interact with the local GP to diagnose prostate cancer at a more curable phase, for example, would not be beneficial without simultaneously optimising the support and advice to men about treatment options and the range of treatments available, while minimising the delays and necessity for travelling long distances. The findings of poorer outcomes for men in a regional area that initiated this research, in company with evidence from our participants, suggests that continuing to work on systemic improvements is an important goal for all those committed to men’s health.

We propose that health data registries can benefit from incorporating qualitative research in their programs. Qualitative data assist in the interpretation of quantitative results, suggest potential avenues of research investigation, reveal and communicate meaning, and show us what a disease entity such as prostate cancer means to the people who experience it.

## Additional files

Additional file 1: Interview Guide 1: Text of the guide used by the interviewer to conduct in-depth interviews with men who had been diagnosed with prostate cancer. (DOC 48 kb)

Additional file 2: Interview Guide 2: Text of the guide used by the interviewer to conduct in-depth interviews with men who had not been diagnosed with prostate cancer. (PDF 170 kb)

## Abbreviation

GP: General Practitioner
PSA: Prostate-specific antigen
PCOR-V: Prostate Cancer Outcome Registry-Victoria
