# Supplementary material for: Men’s perceptions of prostate cancer diagnosis and care: insights from qualitative interviews in Victoria, Australia
Source: BMC Cancer. 2017 Oct 27;17:704. doi: 10.1186/s12885-017-3699-1 (PMC5659031; doi:10.1186/s12885-017-3699-1)
Supplement: Supplementary file 1 — Interview Guide 1: Text of the guide used by the interviewer to conduct in-depth interviews with men who had been diagnosed with prostate cancer. (DOC 48 kb) [file 12885_2017_3699_MOESM1_ESM.doc]

Date: Interviewer:

Identifier: Regional/Metropolitan

**Preamble**

Thank you very much for agreeing to be interviewed. Can I double-check that you have read the Information and Consent Form? Do you have any questions before I begin? If you have not sent a signed consent form, do you consent to participation in this research?

As you know, you were invited to participate in this research so that we can learn more about the experience of being diagnosed with prostate cancer. Everything that you say will be confidential. Your doctor won’t find out anything you say, and nothing you say will affect your care in any way. If we quote you in anything we write, we will take great care to make sure that no-one can identify you or the other men we quote.

**Invitation to tell the prostate cancer story**

I’d like to begin by finding out what this experience has been like for you. Would you tell me your prostate cancer story, please?

*[Where appropriate, encourage elaboration by asking something like, “Please tell me more” or “Can you explain that for me, please?” or “Can you tell me what that was like?”]*

*[The remaining questions are suggestions of topics to initiate if the participant has not already done so.]*

**Before diagnosis**

What led to the diagnosis of prostate cancer?

- What symptoms were troubling you?
- Who suggested the visit to your GP?
- How long was it before you made an appointment?

**Diagnosis**

What did you know about prostate cancer before your diagnosis?

Can you remember what your GP had told you about prostate cancer? Anything about screening? Symptoms?

Please tell me what you remember about being diagnosed.

- Who was with you?
- What did the GP say?
- What was your response? (Feelings, actions.)

**Medical care and support after diagnosis**

What did your GP recommend that you did after the diagnosis?

- Further assessment?
- Treatment options?

How did you feel about the treatment options?

- How did you make a decision about your treatment?
- What did you decide?
- How soon you were able to start treatment?

How did your GP support you during and after treatment?

How did your GP support your family during and after treatment? (Which family members?)

Can you tell me about any symptoms you had?

- Urinary, bowel, sexual?
- How did your GP help you to manage them?

What was good about the care you received from your GP?

What do you think your GP could have done better?

Can you tell me your experiences of a Specialist Prostate Nurse?

Was there any kind of care and support you wish you’d been given?

- How would that have made a difference?

**Choosing a practice or GP**

How long have you been with this practice?

Why did you choose this one?

Do you see a particular doctor?

Why/why not?

Does it vary according to circumstances? Do you make a choice according to your symptoms (or something else)?

Please tell me about what happens when you ring to make an appointment.

Does the receptionist know you? (What is your preference?)

Is it easy to see a GP when you need to?

What led you to make an appointment the last time your rang the doctor?

What makes a good GP for you?

Does a good GP know patients’ families? Why/why not?

Has this been your experience? (A good GP; knowing or not knowing your family.)

**Advice to doctors and policy-makers**

Is early diagnosis of prostate cancer important to you? Why/Why not?

[If yes] What do you think could be done to ensure that prostate cancer is diagnosed as early as possible?

Role of GPs, governments, others?

**Advice to other men**

What advice would you like to give to other men to ensure that they have early diagnosis and treatment?

**Anything we’ve missed?**

Is there anything else you’d like to tell us about prostate cancer and its treatment?

**Demographic information**

We’d like to be able to give a summary of all the men who have talked to us, so I’d like to check some facts with you. None of these will be used to identify you; it’s just so that we can describe things like the average age, and where people come from. Some of these things you’ve told me already, but I might mention some of them just to ensure I’ve noted the details correctly. *[NB Don’t ask about what is very clear from the interview, such as partner status.]*

In what country were you born?

How would you describe your cultural background? *(Prompt if necessary. For example, are you: Aboriginal, Torres Strait Islander, Greek heritage, Vietnamese, … Australian.)*

How old are you now?

How old were you when you were given a diagnosis of prostate cancer?

Can you tell me if you had private health insurance at the time of your diagnosis? (Level of cover; how insurance affected treatment.)

What is your occupation? (If “Retired”: What did you do before you retired?)

Are you in paid employment at the moment? What do you do?

[If he has a partner] Is your partner in paid employment? What does he/she do?

Who lives with you? [Is it just you/you and your partner?]

Do you have family living nearby? Who? Where?

*[Thank the participant for his generous contribution to the research; give/ask where to send a $20 voucher.]*
