# Supplementary material for: Men’s perceptions of prostate cancer diagnosis and care: insights from qualitative interviews in Victoria, Australia
Source: BMC Cancer. 2017 Oct 27;17:704. doi: 10.1186/s12885-017-3699-1 (PMC5659031; doi:10.1186/s12885-017-3699-1)
Supplement: Supplementary file 2 — Interview Guide 2: Text of the guide used by the interviewer to conduct in-depth interviews with men who had not been diagnosed with prostate cancer. (PDF 170 kb) [file 12885_2017_3699_MOESM2_ESM.pdf]

Date:______________________ Interviewer:_______________________

Identifier:___________________ Regional/Metropolitan

Preamble

Thank you very much for agreeing to be interviewed. Can I double-check that you have read the Information and Consent Form? Do you have any questions before I begin? If you have not sent a signed consent form, do you consent to participation in this research?

As you know, you were invited to participate in this research so that we can learn more about men and prostate cancer. Everything that you say will be confidential. If we quote you in anything we write, we will take great care to make sure that no-one can identify you or the other men we quote.

Invitation to tell a prostate cancer story

I'd like to begin by finding out what your experience of prostate cancer is; whether you know anyone who has been diagnosed, whether you have thought about it: anything at all?

[Where appropriate, encourage elaboration by asking something like, "Please tell me more" or "Can you explain that for me, please?" or "Can you tell me what that was like?"]

[The remaining questions are suggestions of topics to initiate if the participant has not already done so.]

Prostate

Have you seen a doctor about your prostate? Why/why not?

Has your doctor spoken to you about your prostate or prostate cancer?

What was said and done? / What do you think your doctor should have done?

What do men you know say about prostate checks or prostate cancer?

What about other people? Your family?

What are your thoughts on your prostate?

What would you recommend as the best way to avoid prostate cancer?

Choosing a practice or GP

How long have you been with your GP practice?

Why did you choose this one?

Do you see a particular doctor? Why/why not?

Does it vary according to circumstances? Do you make a choice according to your symptoms (or something else)?

Please tell me about what happens when you ring to make an appointment.

Does the receptionist know you? (What is your preference?)

Is it easy to see a GP when you need to?

What led you to make an appointment the last time your rang the doctor?

What makes a good GP for you?

Does a good GP know patients' families? Why/why not?

Has this been your experience? (A good GP; knowing or not knowing your family.)

Can you tell me about a time when you wanted to see a doctor and things worked out well?

What about a time when you think you weren't looked after as well as you'd like?

Advice to doctors and policy-makers

Is early diagnosis of prostate cancer important to you? Why/Why not?

[If yes] What do you think could be done to ensure that prostate cancer is diagnosed as early as possible?

Role of GPs, governments, others?

Advice to other men

What advice would you like to give to other men about their prostate health?

<u>Anything we've missed?</u>

Is there anything else you'd like to tell us about the prostate, or prostate cancer and its treatment?

<u>Demographic information</u>

We'd like to be able to give a summary of all the men who have talked to us, so I'd like to check some facts with you. None of these will be used to identify you; it's just so that we can describe things like the average age, and where people come from. Some of these things you've told me already, but I might mention some of them just to ensure I've noted the details correctly. *[NB Don't ask about what is very clear from the interview, such as partner status.]*

In what country were you born?

How would you describe your cultural background? *(Prompt if necessary. For example, are you: Aboriginal, Torres Strait Islander, Greek heritage, Vietnamese, … Australian.)*

How old are you now?

What is your occupation? (If "Retired": What did you do before you retired?)

Are you in paid employment at the moment? What do you do?

[If he has a partner] Is your partner in paid employment? What does he/she do?

Who lives with you? [Is it just you/you and your partner?]

Do you have family living nearby? Who? Where?

[Thank the participant for his generous contribution to the research and give him a $20 voucher.]
